# The promise of paediatric dolutegravir

**DOI:** 10.1002/jia2.25660

**Published:** 2021-01-31

**Authors:** Rachel Golin, Jeffrey M Samuel, B Ryan Phelps, Udita Persaud, Christine Y Malati, George K Siberry

**Affiliations:** ^1^ Office of HIV/AIDS United States Agency for International Development Washington DC USA

**Keywords:** HIV, paediatric, children, antiretroviral therapy, dolutegravir

In 2019, fewer than 55% of the estimated 1.8 million children living with HIV (CLHIV) received life‐saving antiretroviral therapy (ART) [1]. Children in low‐ and middle‐income countries (LMIC) continue to have limited access to optimal paediatric ART [2], and viral load suppression (VLS) rates among children remain unacceptably low [3]. To achieve the UNAIDS 95‐95‐95 benchmarks for all ages by 2030 [4], there is a pressing need for LMIC to have access to robust, affordable and child‐friendly paediatric ART regimens [2].

Since 2018, the World Health Organization (WHO) has recommended the use of dolutegravir (DTG), an integrase strand transfer inhibitor, as part of the preferred first‐line ART regimen for all people living with HIV (PLHIV) for whom there is approved and available dosing [5]. However, to date, DTG has only been available in LMIC as a 50 mg, film‐coated tablet, a formulation that can only be used by children who weigh at least 20 kg [5].

In June 2020, the United States Food and Drug Administration (US FDA) approved a dispersible, 5 mg formulation of DTG for use in infants and children living with HIV [6], and in November 2020, a paediatric DTG 10 mg scored disperisble tablet formulation (DTG10) also received tentative US FDA approval [7]. With these approvals in place, LMIC can expect to have access to generic DTG10 available in 2021 [8].

The introduction of a DTG dispersible tablet is a significant advance in optimal treatment for CLHIV. Based on data extrapolated from studies in adults, DTG’s efficacy is superior to both protease inhibitors (PIs) [9] and non‐nucleoside reverse transcriptase inhibitors (NNRTIs) [10]. Introduction of DTG directly addresses pre‐treatment drug resistance among CLHIV [11] as well as acquired drug resistance after failed NNRTI‐ or PI‐based regimens [8]. Additionally, due to a high genetic barrier to resistance, DTG can be used along with an optimized NRTI backbone as an anchor drug throughout childhood and adulthood [8]. This is especially critical in LMIC where genotypic drug resistance testing is very limited [12].

Paediatric DTG also comes in a convenient, once‐daily, dispersible tablet formulation that can be dissolved and administered alongside dispersible formulations of ABC/3TC [6, 8]. This allows for greater convenience than twice‐daily LPV/r and reduces pill burden [8]. In addition, paediatric DTG overcomes other disadvantages of LPV/r formulations: LPV/r granules and pellets require additional training to ensure appropriate adherence [13]; LPV/r oral solution has poor palatability and requires consistent cold chain until dispensed [13]; and the LPV/r heat‐stable tablet cannot be cut, crushed, chewed, or dissolved [13], precluding the use of this formulation in young CLHIV.

DTG10, which fits into smaller‐sized packaging compared to current paediatric LPV/r formulations, also has the potential to reduce implementation barriers associated with multi‐month dispensing (MMD) of ARVs to children, a practice with well‐documented benefits but slow scale‐up [14]. With DTG10, caregivers will be able to confidently carry and safely store a less bulky, longer lasting supply of paediatric ARVs.

Paediatric DTG is also favourable from a manufacturing standpoint. Challenges with LPV/r production have occurred due to limited use of LPV/r in adults [5] as well as limited manufacturers of paediatric LPV/r formulations [13]. Since DTG has been used in first‐line adult ARV regimens since 2016 [5], the active pharmaceutical ingredient is more readily available. Moreover, dispersible tablets are a common formulation and their scale‐up presents minimal, if any, manufacturing constraints.

Another important paediatric DTG advantage is cost savings. Compared to LPV/r formulations, paediatric programmes can anticipate significant savings in both product and shipping costs. The estimated yearly cost savings of changing a child from LPV/r to DTG10 ranges from $90 to $1,200 USD per child (inclusive of shipping costs), depending on the LPV/r formulation and dosing weight band [15].

In summary, we will soon have an affordable paediatric ARV formulation that has a product profile far better than any preceding paediatric ARV. The advantages of paediatric DTG outlined above are compelling, and the time to plan for DTG10’s rapid introduction and wide‐scale use is now. To adequately prepare for the rapid transition to paediatric DTG, the following is recommended:

1. National HIV treatment guidelines should clearly state that DTG‐based ART is the preferred first‐line and second‐line treatment for all PLHIV, including children who meet the minimum age (four weeks) and weight (three kilograms) thresholds for paediatric DTG [6, 8]. This includes children who are newly initiating HIV treatment, those who are virologically suppressed on non‐DTG regimens, and individuals who experience virological failure on a non‐DTG regimen.
2. Concurrent to revising national guidelines, countries should prepare for product registration with the national medicines regulatory authority, commodity procurement and decentralized distribution while implementing appropriate pharmacovigilance measures, training facility and community cadres and developing/disseminating context‐specific information, education, and communication materials for healthcare workers and caregivers [8].
3. Well‐informed forecasting for DTG should commence, anticipating increased DTG consumption and lower demand for LPV/r. Such forecasts should take into account the number of CLHIV who currently receive ART and the number of anticipated new paediatric infections. As DTG 50 mg can be used in CLHIV who weigh at least 20 kg [5], DTG10 forecasts should be based on CLHIV weighing < 20 kg.
4. Incorporating the elements above, a transition strategy should be jointly agreed upon between supply chain, clinical and other stakeholders, so that countries can place DTG10 orders as early as possible.

ART coverage and viral load suppression in children continue to lag that in adults [3], and paediatric DTG promises to advance epidemic control across this vulnerable group. DTG10, in particular, provides clear advantages for the child, the caregiver and healthcare provider and the broader health system, including the ARV market. A paediatric DTG transition plan that includes targeted national guideline revision, demand anticipation and careful supply chain planning will ensure that individual countries and the global health community can take full advantage of this historic opportunity.

## COMPETING INTERESTS

The authors declare no competing interests.

## AUTHORS’ CONTRIBUTIONS

The initial concept for this commentary was conceived by BRP and CYM. RG, BRP, CYM, and GKS contributed to the initial outline. All authors (RG, JMS, BRP, UP, CYM, and GKS) contributed to the initial manuscript content and revisions. All authors (RG, JMS, BRP, UP, CYM and GKS) reviewed and approved the final commentary prior to publication.
